# Design, synthesis, and biological evaluation of novel 5,6,7-trimethoxy quinolines as potential anticancer agents and tubulin polymerization inhibitors

**DOI:** 10.22038/ijbms.2020.43303.10168

**Published:** 2020-12

**Authors:** Salimeh Mirzaei, Farhad Eisvand, Farzin Hadizadeh, Fatemeh Mosaffa, Razieh Ghodsi

**Affiliations:** 1 Biotechnology Research Center, Pharmaceutical Technology Institute, Mashhad University of Medical Sciences, Mashhad, Iran; 2 Department of Medicinal Chemistry, School of Pharmacy, Mashhad University of Medical Sciences, Mashhad, Iran; 3 Department of Toxicology, School of Pharmacy, Mashhad University of Medical Sciences, Mashhad, Iran

**Keywords:** Anticancer activity, Apoptosis, Quinoline, Resistant cancer cells, Tubulin inhibitors

## Abstract

**Objective(s):**

Microtubules have key roles in essential cellular processes such as mitosis, cell motion, and intracellular organelle transport. Increasing interest has been given to tubulin binding compounds after the introduction of taxanes into clinical oncology. The object of this study was synthesis and biological evaluation of novel 5,6,7-trimethoxy quinolines as tubulin inhibitors.

**Materials and Methods:**

The cytotoxicity of the newly synthesized compounds was assessed against different human cancer cell lines including MCF-7, A2780, MCF-7/MX, A2780/RCIS, and normal cells. Compounds demonstrating the most antiproliferative activity, were chosen to examine their tubulin inhibition activity and their ability to arrest the cell cycle and induce apoptosis. Molecular docking studies and molecular dynamics simulation of compound **7e** in the catalytic site of tubulin were performed.

**Results:**

Most of the synthesized quinolines showed moderate to signiﬁcant cytotoxic activity against human cancer cells. Compounds 7e and 7f, possessing N-(4-benzoyl phenyl) and N-(4-phenoxy phenyl), respectively, exhibited the most antiproliferative activity more potent than the other compounds and exhibited similar antiproliferative activity on both resistant and parental cancer cells.

**Conclusion:**

Flow cytometry analysis of A2780, A2780/RCIS, MCF-7, and MCF-7/MX cancer cells treated with **7e** and **7f** exhibited that these compounds arrested the cell cycle (at the G2/M phase) and induced cellular apoptosis in A2780 cancer cells. These quinolines inhibited tubulin polymerization in a way resembling that of CA-4. Molecular dynamics simulation and molecular docking studies of compound **7e** into the binding site of tubulin displayed the probable interactions of **7e** with the binding site of tubulin.

## Introduction

Microtubule is a fundamental section of the cytoskeleton (1, 2). Microtubules have a key role in many cellular functions, such as motility, division, signaling, and intracellular transport (3, 4) and as a result, the interest to use tubulin as an attractive target has been increased in anticancer drug discovery (5). The microtubule targeting agents MTAs) disrupt microtubule dynamic and arrest cancer cells in the G2/M phase, which results in apoptosis (6). Tubulin inhibitors are divided into three classes (based on their binding sites in tubulin protein) including taxol site inhibitors (e.g., taxol and epothilones) (7), vinblastine site inhibitors (e.g., vinblastine and vincristine) (8), and colchicine site inhibitors (e.g., colchicine) (9). Based on the mechanism of action, these compounds are categorized as microtubule stabilizers such as taxanes and microtubule destabilizers like vinca alkaloids and colchicine (6). Anti-mitotic drugs such as vinca alkaloids and taxanes, have been extensively administrated in the clinical treatment of many human cancers over the past decades (10). Despite the progress in administration of microtubule targeting agents for treatment of patients with cancer, drug resistance and adverse effects such as peripheral neuropathy are the main problems of patients (11-13). This has encouraged medicinal chemists to design and discover the novel antimitotic agents for cancer therapy (14-26). Quinoline derivatives have exhibited strong anticancer activity via a different mechanism of action (27). The natural alkaloid camptothecin and its semisynthetic analog topothecan are two examples of cytotoxic quinolines with significant antitumor activity through inhibition of the DNA topoisomerase I enzyme (28, 29). 2-Styrylquinolines are promising anti-cancer compounds against several cancer cells (30). Recently 2-aryl-trimethoxyquinoline analogs have been reported as tubulin inhibitors (2). Verubulin is a very potent inhibitor of β-tubulin that interacts at or near the colchicine binding site (31).

Molecular hybridization is a useful method to design more potent and new compounds by merging two or more drug pharmacophores into a single compound (32). In this context, inspired by the structures of trimethoxyquinoline and styrylquinoline and therapeutic significance of verubulin, we planned to combine these bioactive scaffolds to develop promising new anticancer agents. 

**Figure 1:**
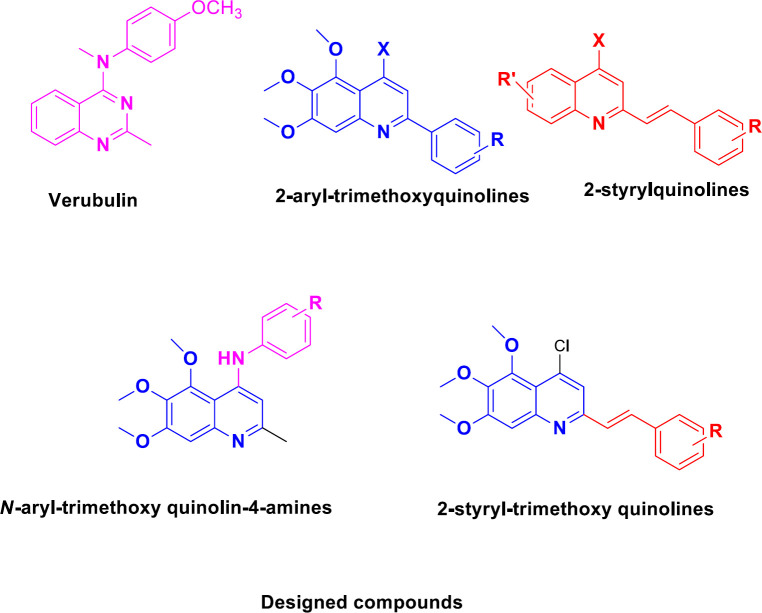
Reported anticancer agents and novel trimethoxy quinolines as tubulin inhibitors

Therefore, we designed and synthesized two series of novel quinoline derivatives possessing trimethoxy quinoline scaffold, one series included N-aryl-trimethoxy quinoline-4-amine derivatives and the other series 2-styryl-trimethoxy quinoline derivatives. The rationale for the design of these quinolines is depicted in Figure 1. The synthesized quinolines were evaluated for their cytotoxic activity against four different human cancer cell lines including MCF-7, MCF-7/MX, A2780, and A2780/RCIS and normal (Huvec) cells. Compounds exhibiting the most cytotoxicity, were evaluated for their potential to induce G2 arrest and apoptosis by flow cytometry. They were also examined for their activity in a microtubule polymerization assay. Moreover, molecular dynamics simulation and docking studies were done to describe the results of biological experiments. 

## Materials and Methods

***Chemistry***

All reagents used in this study were purchased from Merck AG and Aldrich Chemical. Melting points were determined using a Thomas Hoover capillary apparatus. To acquire infrared and NMR spectra, a Perkin Elmer spectrometer (Model 1420) and a Bruker FT-300 MHz instrument were used, respectively. Compounds were solved in Chloroform-D or DMSO-D6 before acquiring NMR spectra. Coupling constant (J) values were assessed in hertz (Hz) and spin multiples were given as s (singlet), d (doublet), t (triplet), q (quartet), or m (multiplet). A 3200 QTRAP LC/MS triple quadrupole mass spectrometer possessing an electrospray ionization (ESI) interface was used to acquire the mass spectra.

***Procedure for synthesis of 2-methylquinolin-4-ol (4) and 4-chloro-2-methylquinoline (5)***

Compounds **4** and **5** were synthesized using the procedure reported in the reference (33).

***General procedure for synthesis of*** ***5,6,7-trimethoxy-2-methyl-N-phenylquinolin-4-amine derivatives (7a-7g)***

Aromatic amine (1 mmol) was added to a solution of compound **5** (1 mmol) and was refluxed in ethanol for 4–12 hr. After completion of the reaction and evaporation of ethanol in vacuum, a yellow precipitate was acquired. Chloroform and n-hexane (70/30) were used to recrystallize the yellow solid.

***5,6,7-Trimethoxy-N-(4-methoxyphenyl)-2-methylquinolin-4-amine (7a)***

Yellow solid; yield: 91%; mp: 191-194 °C; IR (KBr): ν (cm^-1^) 3269 (NH); ^1^H NMR (300 MHz, CDCl_3_) δ (ppm): 2.64 (s, 3H, CH_3_), 3.87 (s, 3H, OCH_3_), 3.93 (s, 3H, OCH_3_), 4.05 (s, 3H, OCH_3_), 4.16 (s, 3H, OCH_3_), 6.17 (s, 1H, Ar-H), 7.01-7.04 ( d, 2H, J=8.7 Hz, Ar-H), 7.22-7.29 (m, 2H, Ar-H), 7.99 (s, 1H, Ar-H), 9.86(s, 1H, NH ), 13C NMR (75 MHz, DMSO-d_6_): 19.86, 55.64, 56.90, 61.37, 62.52, 97.85, 98.43, 104.87, 115.44, 127.46, 128.97, 137.95, 140.53, 149.88, 152.79, 155.87, 158.30, 159.18, LC-MS (ESI): 355.1 (M+H)+.

***N-(3,4-dimethoxyphenyl)-5,6,7-trimethoxy-2-methylquinolin-4-amine (7b)***

**Yellow solid; yield: 89%; mp: 182-185 °**C; ν (cm^-1^) 3261.6 (NH); ^1^H NMR (300 MHz, CDCl_3_) δ (ppm): 2.44 (s, 3H, CH_3_), 3.72 (s, 3H, OCH_3_), 3.75 (s, 3H, OCH_3_), 3.80 (s, 3H, OCH_3_), 3.89 (s, 3H, OCH_3_), 4.04 (s, 3H, OCH_3_), 6.26 (s, 1H, Ar-H), 6.88-6.91 (d, 1H, J=9 Hz, Ar-H), 6.91(s, 1H, Ar-H), 7.01-7.04 (d, 1H, J=9 Hz, Ar-H), 7.40 (s, 1H, Ar-H), 9.95 (s, 1H, NH ), 13C NMR (75 MHz, DMSO-d_6_): 20.29, 56.20, 56.77, 61.49, 62.92, 97.56, 99.39, 105.16, 111.00, 112.71, 118.76, 130.71, 138.82, 140.00, 143.35, 149.96, 150.54, 153.32, 155.12, 157.86. LC-MS (ESI) 385.1 (M+H)^+^

***5,6,7-Trimethoxy*** ***-*** ***2-methyl*** ***-*** ***-N-(3,4,5-methoxyphenyl) quinolin-4-amine (7c)***

Yellow solid; yield: 83%; mp: 175-178 °C; ν (cm^-1^) 3259 (NH); ^1^H NMR (300 MHz, CDCl_3_): δ (ppm): 2.55 (s, 3H, CH_3_), 3.70 (s, 3H, OCH_3_), 3.82 (s, 6H, OCH_3_), 3.88 (s, 3H, OCH_3_), 3.97 (s, 3H, OCH_3_), 4.14 (s, 3H, OCH_3_), 6.48-6.50 (d, 1H, J=6 Hz, Ar-H), 6.82 (s, 2H, Ar-H), 7.52 (s, 1H, Ar-H), 10.15 (s, 1H, NH), 13C NMR (75 MHz, DMSO-d_6_): 19.12, 56.49, 56.63, 56.84, 60.62, 61.54, 63.02, 96.65, 99.73, 104.43, 105.10, 133.34, 137.04, 138.80, 140.13, 150.61, 152.97, 154.08, 155.28, 158.24. LC-MS (ESI) 415.2 (M+H)^+^. 

***2-Methoxy-5-((5,6,7-trimethoxy-2-methylquinolin-4-yl)amino)phenol (7d)***

Yellow solid; yield: 74%; mp: 182-184 °C; ν (cm^-1^) 3265 (NH); 1H NMR (300 MHz, CDCl_3_) δ (ppm): 2.51 (s, 3H, CH_3_), 3.82 (s, 3H, OCH_3_), 3.87 (s, 3H, OCH_3_), 3.96 (s, 3H, OCH_3_), 4.11 (s, 3H, OCH_3_), 3.58 (s, 1H, OH), 6.28-6.30 (d, 1H, J=6 Hz, Ar-H), 6.80-6.83 (d, 1H, J=9 Hz, Ar-H), 6.92 (s, 1H, Ar-H),7.04-7.07 (d, 1H, J=9 Hz, Ar-H), 7.48 (s, 1H, NH), 10.09 (s, 1H, OH). 13C NMR (75 MHz, DMSO-d_6_): 19.63, 56.28, 56.94, 61.51, 62.89, 96.52, 99.20, 104.92, 113.25, 114.29, 117.38, 130.30, 137.63, 140.03, 147.64, 148.05, 150.68, 152.57, 155.69, 158.24. LC-MS (ESI) 371.1 (M+H)^+^

***Phenyl(4-((5,6,7-trimethoxy-2-methylquinolin-4-yl)amino)phenyl-methanone (7e)***

Yellow solid; yield: 65%; mp: 187-190 °C; ν (cm^-1^) 3274 (NH), 1632 (CO); ^1^H NMR (300 MHz, CDCl_3_) δ (ppm): 2.77 (s, 3H, CH_3_), 3.97 (s, 3H, OCH_3_), 4.10 (s, 3H, OCH_3_), 4.22 (s, 3H, OCH_3_), 6.61 (s, 1H, Ar-H), 7.47-7.56 (m, 4H, Ar-H), 7.63-7.65 (m, 1H, Ar-H), 7.83-7.86 (d, 2H, J=9 Hz, Ar-H), 7.98-8.00 (d, 2H, J=6 Hz, Ar-H), 8.11(s, 1H, Ar-H), 10.22 (s, 1H, NH ), 13C NMR (75 MHz-CDCl_3_-d6): 20.27,57.04, 61.46, 62.74, 98.18, 98.92, 105.39, 124.22, 128.52, 129.95, 132.19, 132.87, 136.30, 137.06, 138.16, 140.45, 141.05, 149.55, 153.52, 153.91, 158.63, 195.19. LC-MS (ESI) 429.1 (M+H)^+^

***5,6,7-Trimethoxy-2-methyl-N-(4-phenoxyphenyl)quinolin-4-amine (7f)***

Yellow solid; yield: 68%; mp: 192-195 °C; ν (cm^-1^) 3278 (NH), ^1^H NMR (300 MHz, CDCl_3_) δ (ppm): 2.51 (s, 3H, CH_3_), 3.88 (s, 3H, OCH_3_), 3.96 (s, 3H, OCH_3_), 4.12 (s, 3H, OCH_3_), 6.36 (s, 1H, Ar-H), 7.09-7.12 (d, 1H, J=9 Hz, Ar-H), 7.17-7.22 (m, 3H, Ar-H), 7.45-7.50 (m, 5H, Ar-H), 10.13(s, 1H, NH),13C NMR (75 MHz, DMSO-d_6_): 20.13, 56.80, 62.51, 62.92, 119.09, 119.35, 120.04, 120.18, 124.32, 126.05, 128.65, 130.58, 130.66, 133.13, 140.10, 150.54, 153.30, 154.98, 156.06, 156.81, 158.02. LC-MS (ESI) 417.2 (M+H)^+^

***5,6,7-Trimethoxy-2-methyl-N-(2-phenoxyphenyl)quinolin-4-amine (7g)***

Yellow solid; yield: 61%; mp: 150-153 °C; ν (cm^-1^) 3272 (NH), ^1^H NMR (300 MHz, CDCl_3_) δ (ppm): 2.54 (s, 3H, CH_3_), 3.83 (s, 3H, OCH_3_), 3.89 (s, 3H, OCH_3_), 3.93 (s, 3H, OCH_3_), 6.48-6.50 (d, 1H, J=6 Hz, Ar-H), 6.91-6.94 (d, 1H, J=9 Hz, Ar-H), 6.98-7.01 (d, 2H, J=6 Hz, Ar-H), 7.08-7.15 (m, 2H, Ar-H), 7.23-7.45 (m, 4H, Ar-H), 7.65-7.68 (d, 1H, J=9 Hz, Ar-H), 10.12 (s, 1H, NH). LC-MS (ESI) 417.2 (M+H)^+^

***General procedure for the synthesis of E-4-chloro-5,6,7-trimethoxy-2-styryilquinoline derivatives (9a-9g)***

Compound **5** (1 mmol), para toluene sulfonamide (1 mmol) and different benzaldehydes (1 mmol) were refluxed in toluene at 140 °C (24–48 hr) then chloroform was added to the mixture. The solvents were evaporated in vacuum, the obtained precipitate was filtrated and recrystallized (ethanol) to obtain pure compounds (33).

***E-4-Chloro-5,6,7-trimethoxy-2-styrylquinoline (9a)***

White solid; yield: 60%; mp: 110-113 °C; ^1^H NMR (300 MHz, CDCl_3_): δ (ppm) 3.90 (s, 3H, OCH_3_), 3.92 (s, 3H, OCH_3_), 3.94 (s, 3H, OCH_3_), 7.13-7.18 (d, 1H, J=15 Hz, CH=CH), 7.21(s, 1H, Ar-H), 7.25-7.27 (d, 1H, J=6 Hz, Ar-H), 7.29-7.34 (m, 2H, Ar-H), 7.46 (s, 1H, Ar-H), 7.52-7.57(m, 3H, Ar-H), ^13^C NMR (75 MHz-CDCl_3_-d6): 29.73, 56.11, 61.29, 61.93, 105.41, 115.83, 119.88, 127.30, 127.57, 128.84, 134.74, 136.25, 139.58, 143.21, 147.84, 148.18, 154.90, 156.05. LC-MS (ESI) 378 (M+Na)^+^

***E-4-Chloro-5,6,7-trimethoxy-2-(4-methylstyryl)quinoline (9b)***

White solid; yield: 65%; mp: 153-155 °C; ^1^H NMR (300 MHz, CDCl_3_) δ (ppm): 2.35 (s, 3H, CH_3_), 3.89 (s, 3H, OCH_3_), 3.90(s, 3H, OCH_3_), 4.00 (s, 3H, OCH_3_), 4.03(s, 3H, OCH_3_), 7.24 (s, 1H, Ar-H), 7.27-7.33 (dd, 3H), 7.60-7.62 (d, 2H, J=6 Hz, Ar-H), 7.76 (s, 1H, Ar-H), 7.79-7.84 (d, 1H, J=15 Hz, CH=CH). LC-MS (ESI) 392 (M+Na)^+^

***E-4-Chloro-5,6,7-trimethoxy-2-(4-fluorostyryl)quinoline (9c)***

White solid; yield: 73%; mp: 173-176 °C; ^1^H NMR (300 MHz, CDCl_3_) δ (ppm): 3.89(s, 3H, OCH_3_), 3.90 (s, 3H, OCH_3_), 4.00 (s, 3H, OCH_3_), 7.25-7.29 (m, 2H, Ar-H), 7.31-7.35 (m, 2H, Ar-H), 7.75-7.80 (m, 3H, Ar-H), 7.81-7.87 (d, 1H, J=18 Hz, CH=CH), ^13^C NMR (75 MHz, DMSO-d_6_): 56.51, 61.31, 62.21, 105.89, 115.18, 116.30 (d, *J*_C, F _= 21 Hz),120.64, 127.51, 129.75, 129.86, 133.11, 133.15, 138.78, 143.26, 147.68, 147.88, 155.64 (d, *J*_C, F _= 100.5 Hz). LC-MS (ESI) 396 (M+Na)^+^

***E-4-Chloro-5,6,7-trimethoxy-2-(4-methoxystyryl)quinoline (9d)***

White solid; yield: 67%; mp: 125-127 °C; ^1^H NMR (300 MHz, CDCl_3_) δ (ppm): 3.82(s, 3H, OCH_3_), 3.89(s, 3H, OCH_3_), 3.90(s, 3H, OCH_3_), 4.00 (s, 3H, OCH_3_), 7.00-7.03 (d, 2H, J=9 Hz, Ar-H), 7.20-7.25 (d, 1H, J=15 Hz, CH=CH), 7.32 (s, 1H, Ar-H), 7.66-7.69 (d, 2H, J=9 Hz, Ar-H), 7.76 (s, 1H, Ar-H), 7.78-7.84 (d, 1H, J=15 Hz, CH=CH), 13C NMR (75 MHz, DMSO-d_6_): 55.71, 56.51, 61.30, 62.19, 105.71, 114.21, 114.85, 114.97, 120.50, 125.18, 129.14, 129.30, 131.15, 134.83, 138.71, 143.07, 147.67, 147.97, 155.39, 156.29, 160.43; LC-MS (ESI) 378 (M+H)^+^.

***E-4-Chloro-5,6,7-trimethoxy-2-(3,4-dimethoxystyryl)quinoline (9e)***

White solid; yield: 58%; mp: 122-124 °C; ^1^H NMR (300 MHz, CDCl_3_) δ (ppm): 3.78 (s, 3H, OCH_3_), 3.82(s, 3H, OCH_3_), 3.87 (s, 3H, OCH_3_), 3.90(s, 3H, OCH_3_), 4.00 (s, 3H, OCH_3_), 7.0-7.03 (d, 1H, J=9 Hz, Ar-H), 7.22-7.30 (m, 3H, Ar-H), 7.36 (s, 1H, Ar-H), 7.74 (s, 1H, Ar-H), 7.77-7.82 (d, 1H, J=15 Hz, CH=CH), 13C NMR (75 MHz, DMSO-d_6_): 55.97, 56.00, 56.48, 61.31, 62.20, 105.82, 110.17, 110.24, 112.21, 114.95, 120.52, 121.68, 125.43, 129.42, 135.14, 138.61, 143.06, 147.94, 149.47, 150.22, 155.46, 156.26. LC-MS (ESI) 438.1 (M+Na)^+^

***E-4-Chloro-5,6,7-trimethoxy-2-(3,4,5-trimethoxystyryl)quinoline (9f)***

White solid; yield: 62%; mp: 163-165 °C; ^1^H NMR (300 MHz, CDCl_3_) δ (ppm): 3.92(s, 3H, OCH_3_), 3.95(s, 6H, OCH_3_), 4.01 (s, 3H, OCH_3_), 4.03(s, 3H, OCH_3_), 4.05(s, 3H, OCH_3_), 6.89 (s, 2H, Ar-H), 7.16-7.22 (d, 1H, J=18 Hz, CH=CH), 7.30 (s, 1H, Ar-H), 7.53-7.58(d, 1H, J=15 Hz, CH=CH), 7.60 (s, 1H, Ar-H), 13C NMR (75 MHz-CDCl_3_-d6): 56.14, 61.01, 61.28, 61.94, 104.32, 105.34, 115.18, 119.49, 127.12, 131.87, 134.64, 138.91, 139.63, 143.22, 147.82, 148.24, 153.47, 154.87, 156.11. LC-MS (ESI) 468.1 (M+Na)^+^

***E-4-Chloro-5,6,7-trimethoxy-2-(4-nitrostyryl)quinoline (9g)***

White solid; yield: 68%; mp: 162-164 °C; ^1^H NMR (300 MHz, CDCl_3_) δ (ppm): 4.02 (s, 3H, OCH_3_), 4.05(s, 3H, OCH_3_), 4.07 (s, 3H, OCH_3_), 7.30-7.34 (d, 1H, J=12 Hz, Ar-H), 7.37-7.42 (d, 1H, J=15 Hz, CH=CH), 7.57 (s, 1H, Ar-H), 7.71-7.78 (m, 3H, Ar-H), 8.28-8.31(d, 2H, J=9 Hz, Ar-H), 13C NMR (75 MHz, CDCl_3_-d6): 56.18, 61.31, 61.96, 105.41, 116.39, 120.38, 124.24, 127.72, 129.73, 131.72, 132.00, 140.02, 142.66, 143.75, 147.50, 148.17, 153.48, 156.37. LC-MS (ESI) 423.1 (M+Na)^+^

***Biological evaluation***

*Antiproliferative activity assay*

The MTT assay was done by seeding 5.0×10^3^ human cancer cells or normal cells per well in 96-well plates (34-36). Following overnight incubation of the cells in 5% CO2 at 37 °C, culture medium of each well was exchanged with medium having reference anti-cancer agent CA-4 as well as different concentrations of synthesized compounds and RPMI (no drug) negative control and cells were incubated 48 hr. Then MTT solution (25 μl, 4 mg ml ^-1^) was added to each well and the cells were incubated at 37 °C (for 3 hr). Finally, formazan crystals were dissolved in DMSO (100 μl) and plates were read in a plate reader (Synergy H4, USA) at 540 nm. 

*In vitro tubulin polymerization assay*

 A commercial kit (cytoskeleton, cat. #BK011P) was used to evaluate the tubulin polymerization inhibition activity of compounds (37). Briefly, tubulin protein was added to tubulin buffer (80 mM PIPES, 2 mM MgCl2, 0.5 mM EGTA, 1 mM GTP, 60% (v/v) glycerol, PH 6.9) and the acquired mixture was added to wells of a 96-well plate comprising the cytotoxic compounds or vehicle. Tubulin polymerization was observed by detecting the fluorescence augmentation due to the addition of a fluorescence reporter into microtubules as polymerization occurred. Polymerization was measured by excitation at 360 nm and emission at 420 nm for 1 hr at 1 min intervals in a plate reader (Synergy H4, USA). CA-4 (5 μM) and paclitaxel (3 μM) were used as positive destabilizing and positive stabilizing controls, respectively.

*Cell cycle analysis using flow cytometry*

Flow cytometry with propidium iodide (PI) was used to analyze the cell numbers in the cell cycle phases. Briefly cancer cells (2.5 x 105) were seeded to 6-well cell culture plates and incubated (24 hr), then different concentrations of the compounds **7e** and **7f** and vehicle alone were added to the cells and incubated (48 hr), then washed with PBS and ﬁxed with 70% ethanol, then twice washed with PBS and incubated at 37 °C (0.5 hr) in a PBS solution possessing 0.1 mg/mL RNase A and propidium iodide. Flowjo software 7.6 was used to analyze the data regarding the cell numbers in different phases of the cell cycle. 

*Apoptosis analysis*

A2780 cells (2.5 x 105) were seeded in six-well plates and incubated overnight. Then the medium was replaced with a complete medium containing compound **7e** (3 and 15 µM concentrations). After 24 hr incubation, the cells were seeded and washed with PBS and incubated with buffer including 10 mM HEPES, 140 mM NaCl, and 2.5 mM CaCl2 (pH 7.4) and also PI and annexin V-FITC at room temperature (20 min) (38). The samples were analyzed using a flow cytometer (Beckman Coulter), and the results were assessed using the Flowjo software.

***Molecular modeling***

2D structure of the compounds was prepared in Chem Draw Ultra 8.0 software and 3D structure was prepared by Hyperchem 7.0 software. The X-ray crystal structure of tubulin (PDB ID: 1SA0) was downloaded from PDB (Protein Data Bank, www.rcsb.org). Further changes (addition of polar hydrogen or deletion of water molecules) were done by MOE software. Synthesized ligands were docked into the colchicine binding site of tubulin by MOE software. The top-score docking poses were chosen for ligand–target interaction analysis using the LigX module in MOE Software. 

***Molecular dynamic simulation***

In order to find the interaction between protein and ligand at the actual natural situation, molecular dynamic simulation studies were done. Calculations were completed by NAMD 2.12 program (www.ks.uiuc. edu/Research/namd) with CHARMM27 force field. To assess the results Visual Molecular Dynamics (VMD) (www.ks.uiuc.edu/ Research/vmd) was used. The force field parameters of compound **7e **were obtained using Swiss Param (http:// swissparam.chr). Whole structures were occupied in the center of a TIP3 water box (with dimensions 93.218 Å × 94.341 Å × 94.117 Å), using the VMD program. The systems were neutralized by adding chloride and sodium ions. The Particle-Mesh Ewald (PME) algorithm with a grid spacing of 1A° and periodic boundary conditions were used. A cut-off 15 (Å) was used to the short-range Lennard-Jones interactions. Finally, MD simulations were done with a time step of 2 fs for 100 ns (ns). The trajectory of the system was stored at every 1ps and examined by VMD analyzer Tools. A system with an AMD Ryzen Thread ripper 1950X 16- Core, 3.40 GHz processor and 32.0 GB installed memory (RAM) configuration was used in this study (21). 

## Results

***Synthesis***

Compounds **4** and **5** were synthesized using the procedure reported in references (33, 39), compound **5** reacted with aromatic amines (**6)** in ethanol to afford target 5,6,7-trimethoxy-2-methyl-N-phenylquinolin-4-amine derivatives (**7)**. Compound **5** with aromatic aldehydes, **8a-8g,** and para toluene sulfonamide were refluxed in toluene at 140 °C to obtain target (E)-4-chloro-5,6,7-trimethoxy-2-styryiquinoline derivatives (**9)** (40).The structures of compounds were characterized using NMR (nuclear magnetic resonance) and mass spectrometry.

***Biological evaluation***

*In vitro anticancer activity*

The cytotoxic activity of the synthesized compounds was evaluated against different human cancer cell lines including A2780, A2780/RCIS, MCF-7 and MCF-7/MX, and normal cells by MTT assay. Combretastatin A-4 (CA-4) was used as the positive control. The results were shown in Table 1. Some of the tested compounds (**7e**, **7f,** and **7g**) exhibited cytotoxic activity with IC_50_ values ranging from 5.02-35.75 μM against all four human cancer cells.

ClogP values of compounds also were measured (Table 1). Compounds with more ClogP values (**7e**,** 7g,** and **7f**), showed more cytotoxicity in comparison with those with less ClogP. Calculated logP (Clog P) is an important factor in membrane permeability and hence anticancer activity.

*Tubulin polymerization assay*

In order to check if the cytotoxic activity of our quinolines was correlated to their ability to inhibit tubulin, compounds **7e** and **7f** and CA4 (polymerization suppressor) and paclitaxel (polymerization promoter) were assessed for their activity in a microtubule polymerization assay.

These quinolines at the concentration of 20 µM inhibited tubulin polymerization in a way similar to that of CA-4 (5 µM). Compound **7e** inhibited tubulin polymerization more than **7f**. The benzoyl group in compound **7e** may increase its afﬁnity to bind to tubulin, compared with the phenoxy group in compound **7f**.

*Cell cycle analysis using* *ﬂ* *ow cytometry*

Tubulin inhibitors arrest cell cycle at the G2/M phase in cancer cells owing to destruction of the microtubular cytoskeleton. To get more understanding of the mechanism of action, the effects of compounds **7f** and **7e** on the cell cycle were examined by ﬂow cytometry in four human cancer cell lines (MCF-7, MCF-7/MX, A2780, and A2780/RCIS). As shown in Figure 3, this assay revealed that **7e** and **7f** resulted in cell-cycle arrest at the G2/M phase. When A2780 cancer cells were treated with **7f** and **7e** for 48 hr, the percentage of cells in the G2/M phase was 15.70% (**7f**, 1 μM), 17.74% (**7f**, 10 μM), 23.30% (**7e**, 5μM), and 4.15% (**7e**, 15μM) compared with the negative control (4.84%). Moreover, the percentage of A2780 cancer cells in the subG1 phase was 18.58% (**7f**, 10 μM) and 68.16% (**7e**, 15 μM). The results proved that **7e** and **7f**, induced apoptosis in A2780, which is in good agreement to their cytotoxic activity as well.

Compound **7f** at concentrations of 1μM and 5 μM caused G2/M arrest in 20.68% and 19.38% of A2780/RCIS cancer cells, respectively. When A2780/RCIS cancer cells were treated with **7e **at concentrations of 1 μM and 5 μM**, **24.91% and 25.93% of cells arrested at the G2/M phase, respectively, compared with the negative control (9.09%). In untreated MCF-7 cancer cells, 3.49 % of the cells were at the G2/M phase, while in MCF-7 cells treated with **7f** at concentrations of 1μM and 10 µM, the percentage of the cells in the G2/M phase increased to 19.01% and 24.01%, respectively. Compound **7e** at concentrations of 20 μM and 30 μM caused G2/M arrest in 25.91% and 34.45% of MCF-7 cancer cells, respectively. In MCF-7/MX cancer cells, the percentage of cells at the G2/M phase from 3.42% in the control group, increased to 23.73% and 29.37% following treatment with **7f** at concentrations of 10 µM and 20 µM, respectively. The percentage of cells arrested at the G2/M phase by **7e** in MCF-7/MX cancer cells increased from 3.12% (control group) to 25.41 % and 37.85% at concentrations of 1 µM and 10 µM, respectively. The results proved that **7e** and **7f**, can exert their cytotoxic activity through cell cycle arrest at the G2/M phase as well.

*Apoptosis assay (Annexin V binding staining)*

 As mentioned above compound **7e **proved to be an apoptosis inducer in A2780 cancer cells. Then, to investigate the mode of cell death caused by **7e**, flow cytometry was done using propidium iodide (PI) (19), which only colors DNA and only enter dead cells, and Annexin-V (the fluorescence probe), which binds to the early apoptotic cells selectively (20). When A2780 cells were treated with compound **7e** at the concentrations 3 µM and 15 µM for 24 hr, the results showed an accumulation of total apoptotic cells including early and late apoptotic cells from 3.09% (in untreated control) to 8.02 % and 28.57 %, respectively (Figure 5). These results revealed that compound **7e **probably exerted its anti-cancer effect through induction of cellular apoptosis.

***Molecular modeling (docking) studies***

In order to explore the interactions of quinolines in the catalytic site of tubulin (PDB ID: 4O2B), docking studies were performed by MOE 2015.10. The best pose of each ligand was extracted from 100 generated top poses (free energy of binding). The binding manner of the most cytotoxic compounds **7e**, **7f,** and **7g** were shown in Figure 6. Hydrogen bonds and hydrophobic interactions were detected by residues such as Leuβ 248, Serα 178, Lysβ 352, Asnα 101, Gluα 183, and Asnβ 258 with these compounds. The 2D representation of the interaction between compound **7e** in complex with tubulin was shown in Figure S1. The hydrogen bonding interactions were seen between the methoxy groups of compound **7e** with residue Lysβ 254 and Asnα 101 and also nitrogen atom of the quinoline ring could form a hydrogen bonding interaction with Gln 11α. There was also arene-cation interaction with residue Lysβ 254 which improved the binding affinity of compound 7e to tubulin. The reference compound CA-4 with binding energy of -13.84 Kcal/mol formed four hydrogen bonds with residues Lysβ 254, Tyr 224α, Asn 258β, and Gln 11α (Figure S2). Docking studies showed that the methoxy groups of compound **7e **and reference compound CA-4 formed hydrogen bonding interactions with residue Lysβ 254. As mentioned above, compound **7e**, possessing benzoyl substituent exhibited the most potent inhibitory effects and the lowest binding energy (∆G= -12.987 Kcal/mol, which is less than that of CA-4) in molecular modeling study and used for additional MD simulation studies.

***Molecular dynamic (MD) simulation***

In order to explain the behavior of protein upon binding to inhibitor, MD simulation has been performed. The conformational variations in ligand-bond protein were compared with apo-form of tubulin by RMSD and RMSD per residue plots for 80 ns simulations (41, 42). Figure 7 exhibited that the RMSD of backbone (Cα, C, and N) of apo-form and ligand-bond protein **7e** reached stability after about 10 ns of simulation. The average RMSD value of ligand-bond protein was 1.029±0.108 A°, whereas the apo-form was with an average RMSD value of 1.54±0.281 A°. In the RMSD of apo-form, the change was greater than the ligand-bond protein. The RMSD per residue plots of apo-form and ligand-bond protein were illustrated in Figure 8. The average RMSD per residue of ligand-bond protein and apo-form were 0.998±0.528 and 1.120±0.525 A°, respectively. The relatively lower RMSD per residue values of ligand-bond protein could be described by the stronger binding of the ligand with residues located in the binding site that led to their lower flexibility. As presented in Figure 9, Rg values of the apo-form and ligand-bond protein **7e** were 30.44±0.157 and 30.36±0.174 A° (mean±SD), respectively. The graphs exhibited that the Rg of ligand-bond protein was increased for the first 20 ns of simulation and then endured constant throughout the simulation run. This reveals the stabilization of the ligand-bond protein.

Hydrogen bonds have a vital role in the stability of the complex of ligand and protein. This stability of the hydrogen bonds was due to the existence of the ligand in the protein binding site (Figure 10). The findings showed that ligand formed 2-3 hydrogen bonds with protein. These hydrogen bonds improved the binding attraction of ligand with protein. The mean of intramolecular hydrogen bonds of protein was 201.43±11.49. These hydrogen-bond networks stabilized the secondary structure of protein (Figure 11). The ligand bond protein potential energy was found to be -167697±229.038 kcal/mol indicating the stability of the system. Interaction between compound **7e** in the crystal structure of tubulin (PDB ID: 4O2B) was studied after 80 nsec molecular dynamic simulation by LigX in MOE (Figure 12).

## Discussion

In the current study, a new series of 5,6,7-trimethoxy-2-methyl-N-phenylquinolin-4-amine derivatives and (E)-4-chloro-5,6,7-trimethoxy-2-styryiquinoline analogs were synthesized using different reactions. *In vitro* antiproliferative activities of the synthesized compounds were evaluated against four human cancer cell lines including MCF-7, MCF-7/MX, A2780, A2780/RCIS, and normal (Huvec) cell line by MTT assay. Among them, **7e** and **7f** possessing N-(4-benzoyl phenyl) and N-(4-phenoxyphenyl), respectively, showed significant cytotoxic activity against all four human cancer cells.

Generally, N-aryl-trimethoxy quinolin-4-amine derivatives showed more cytotoxicity in cancer cells compared with 2-styryl-trimethoxy quinoline derivatives. These compounds also showed more cytotoxicity on A2780 cancer cells in comparison with the other three cell lines. Among the synthesized quinolines, compounds **7f** and **7e**, possessing N-(4-phenoxyphenyl) and N-(4-benzoyl phenyl), respectively, demonstrated the most potent antiproliferative activity, and interestingly showed higher cytotoxicity against resistant cancer cells (A2780/RCIS and MCF-7/MX) in comparison with their parental cells (A2780 and MCF-7). However, 2-styryl-trimethoxy quinoline derivatives except for **9a** and **9d**, did not display significant cytotoxic activity at concentrations below 100 μM.

Inserting methoxy groups in positions 3 and 5 of N-phenyl moiety of N-aryl-trimethoxy quinolin-4-amine derivatives, decreased the cytotoxicity (compare the cytotoxic activity of compounds **7a**-**7c**). Replacing the methoxy group of **7a** with phenoxy group increased the cytotoxicity significantly (compare the cytotoxic activity of compounds **7a** and **7f**). This may be due to the more lipophilicity of the phenoxy group. Replacing the benzoyl group of **7e** with phenoxy group increased the cytotoxicity slightly (compare the cytotoxic activity of compounds **7e** and **7f**). 

Generally, in N-aryl-trimethoxy quinolin-4-amines, the cytotoxicity of compounds increased with increasing the lipophilicity of substitution in position 4 of the N-phenyl group. Compounds with substitutions of phenoxy and benzoyl showed more cytotoxic activity in comparison with the other quinolines. As the cytotoxicity of **7f** is more than its isomer, **7g**, the position of the phenoxy group is also important in the cytotoxic effect of N-aryl-trimethoxy quinolin-4-amine derivatives. In 2-styryl-trimethoxy quinoline derivatives, compound **9f**, possessing 3,4,5-trimethoxystyryl moiety demonstrated the most significant cytotoxic effect in A2780/RCIS and MCF-7/MX cancer cells and interestingly, these resistant cancer cells were more sensitive to the cytotoxicity of **9f**, compared with parental cells (A2780 and MCF-7). Replacing the methoxy group of **9d** with flour, methyl and nitro groups decreased the cytotoxicity (compare the cytotoxic activity of compounds **9d**, **9b**, **9c,** and **9g**). None of the quinolines, except **7f** and **7g,** displayed major cytotoxicity on normal Huvec cell line. Compound **7e** showed potent and selective cytotoxic effects in cancer cells. The cell cycle analysis indicated that **7e** and **7f** disrupted the microtubule network and arrested cells in the G2/M phase of cell cycle in all four human cancer cell lines. Annexin V binding staining studies on A2780 cancer cells showed that compound **7e **induced cell apoptosis in a concentration-dependent manner. These results confirmed that compound **7e **possibly exerted its anti-proliferative effect through induction of cellular apoptosis. Compounds **7e** and **7f **also inhibited tubulin polymerization in a manner similar to that of CA-4. The binding manner of compounds **7e**, **7f,** and **7g** in the binding site of tubulin displayed a possible mode of interaction between this compound and tubulin. Compound **7e**, possessing benzoyl substituent exhibited the most potent inhibitory effects and the lowest binding energy (∆G= -12.987 Kcal/mol), in molecular modeling study and used for additional MD simulation studies. Molecular dynamics simulation showed that compound **7e** interacts with tubulin by making hydrogen bonds. Also, lower RMSD, RMSD per residue, and Rg (radius of gyration) values of ligand-bond protein than apo-form indicated reduction of conformation flexibility and stability of protein during the simulation.

**Figure 2:**
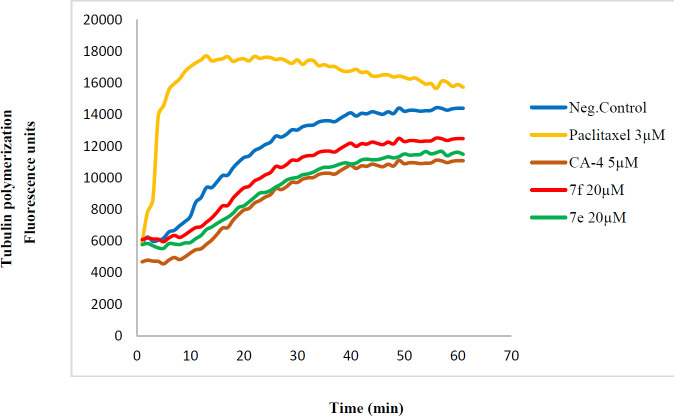
Effect of compounds 7e and 7f on *in vitro* tubulin polymerization

**Scheme 1:**
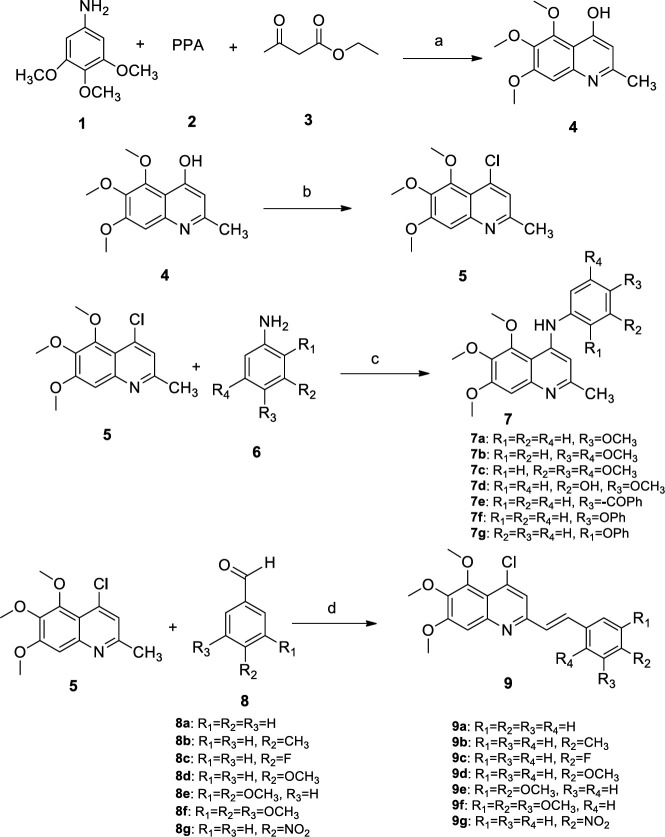
Reagents and conditions: (a) 120° C, 2 hr (b) POCl3, 120 oC, 2 hr (c) absolute ethanol, reflux, 4–12 hr, (d) para toluene sulfonamide, toluene, 140 °C, reflux

**Table 1:** *In vitro* antiproliferative activities (IC50a (µM)) of quinolines and CA-4 against human cancer cell lines

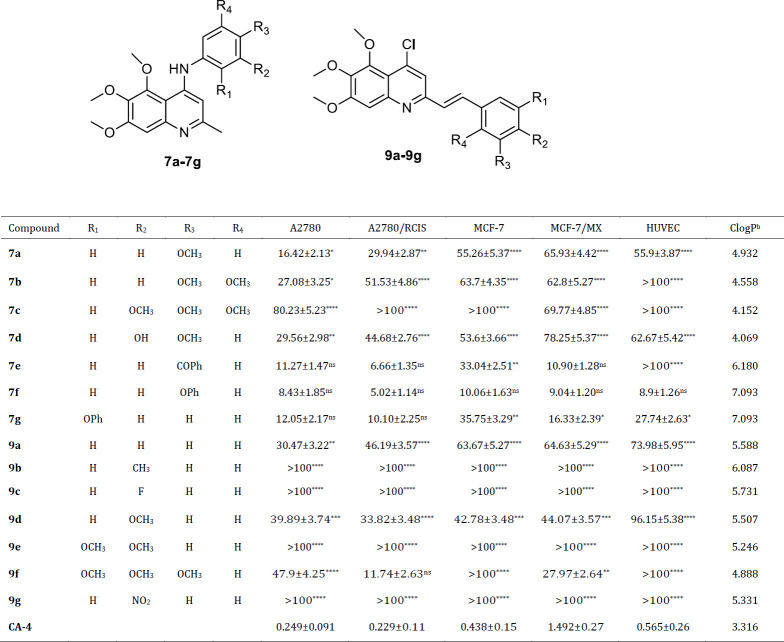

**Figure 3:**
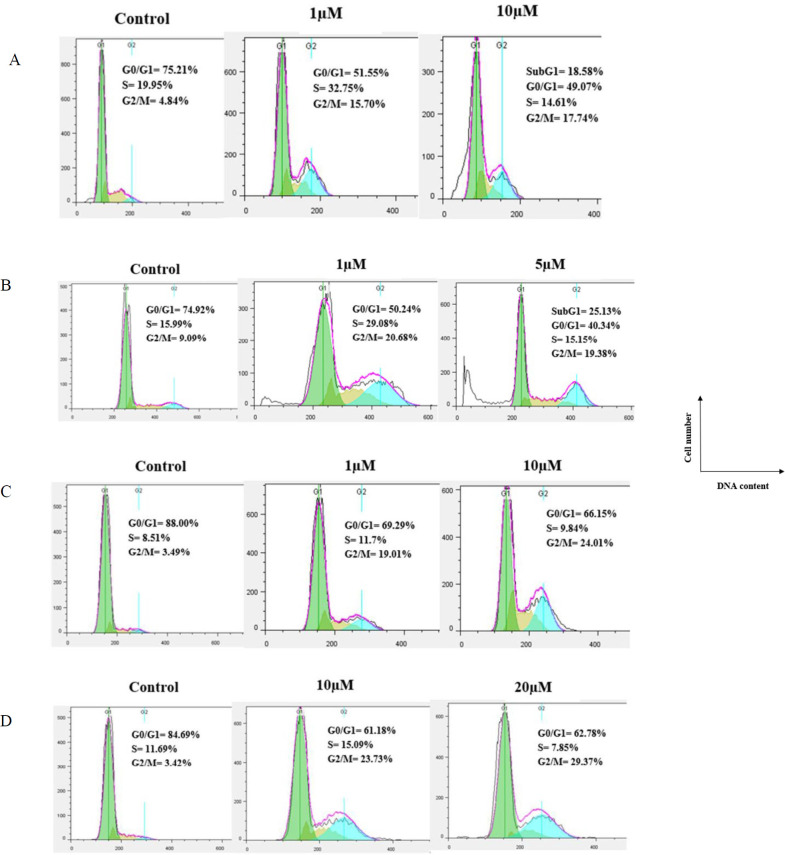
Flow cytometry analysis of compound 7f, in human cancer cell lines: (A) A2780, (B) A2780/RCIS, (C) MCF-7, (D) MCF-7/MX

**Figure 4.**
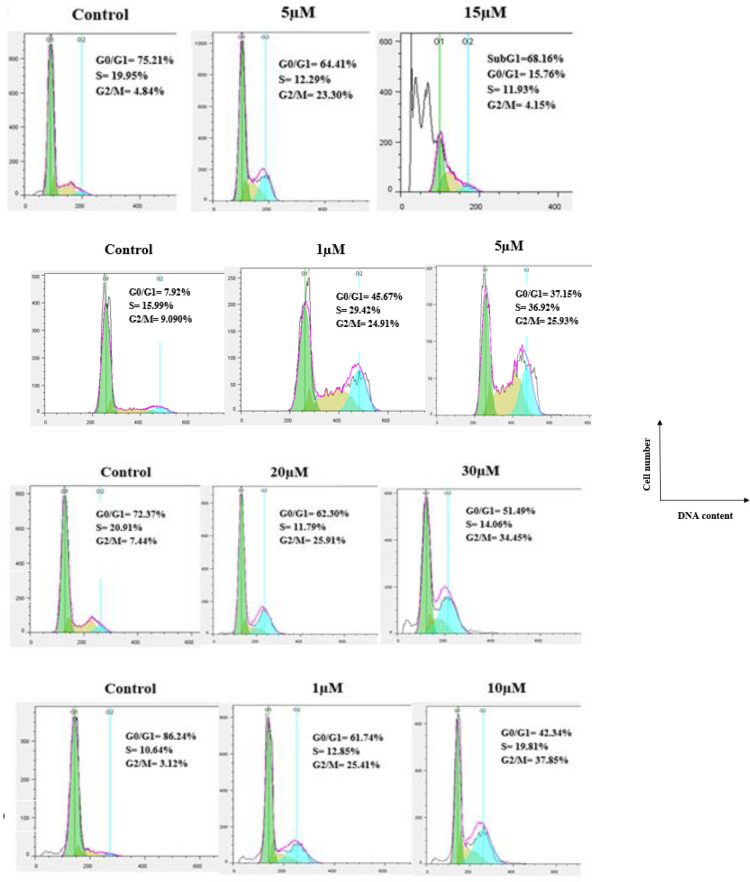
Flow cytometry analysis of compound 7e in human cancer cell lines, (A) A2780, (B) A2780/RCIS, (C) MCF-7, (D) MCF-7/MX

**Figure 5:**
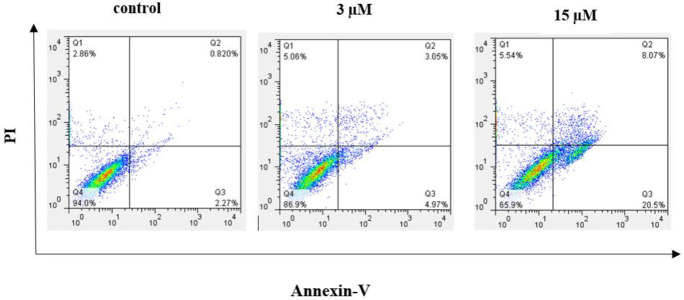
Effect of 7e on the apoptosis of A2780 cancer cells assessed using Annexin V/PI double staining test by flow cytometry. The percentages of cells in each stage of apoptosis were quantitated by flow cytometry

**Figure 6:**
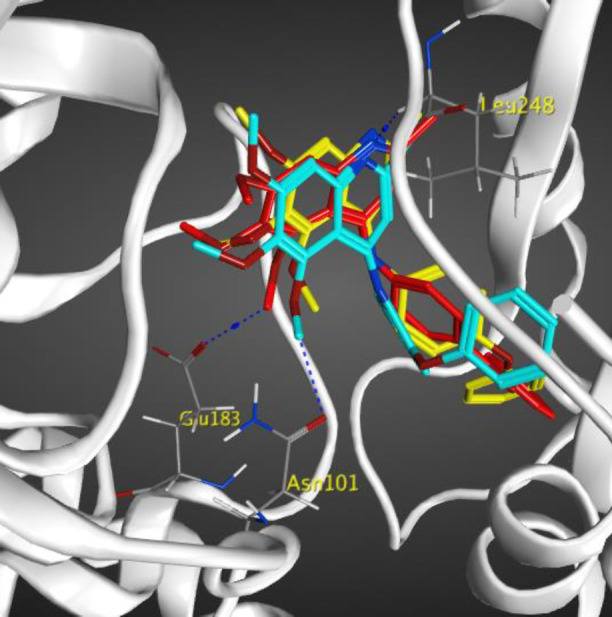
Compounds 7e in red, 7f in yellow, and 7g in blue docked in the active site of tubulin

**Figure 7:**
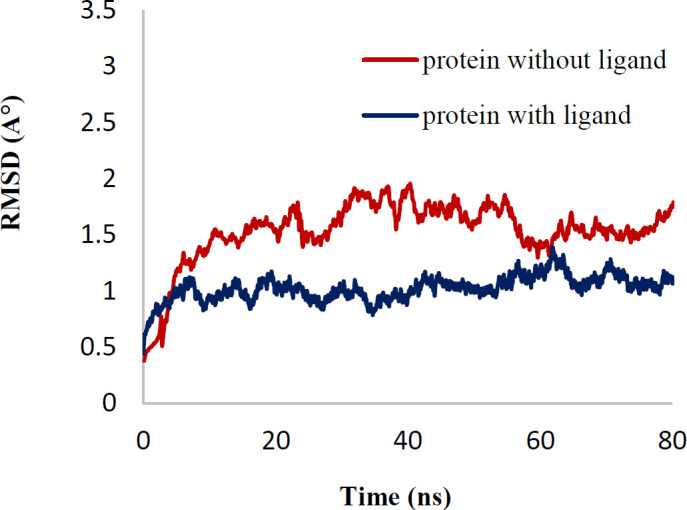
RMSD between protein with ligand and without ligand

**Figure 8:**
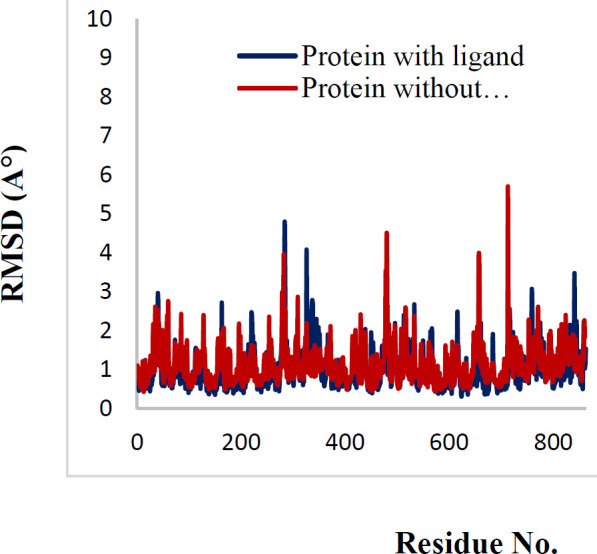
RMSD per residue of protein with ligand and without ligand during 80 nsec simulation

**Figure 9:**
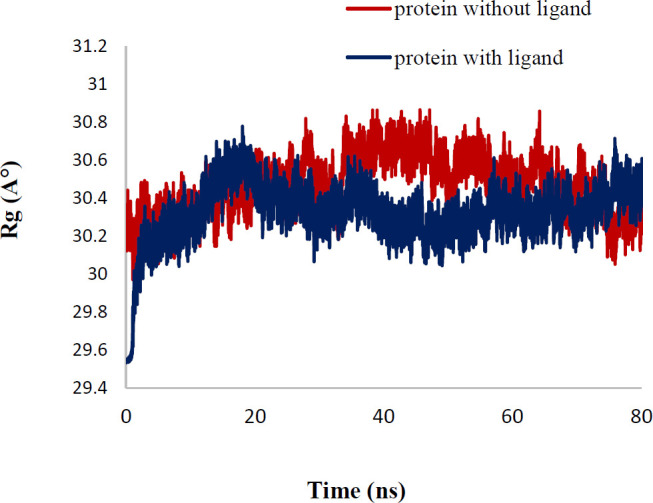
Rg of protein with ligand and without ligand

**Figure 10:**
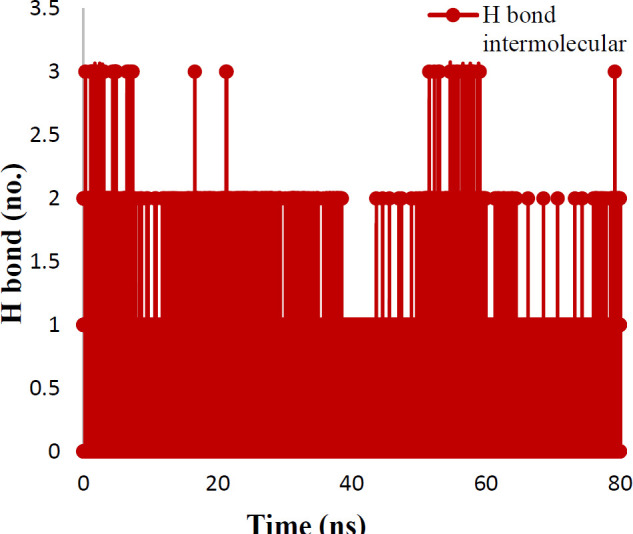
Number of hydrogen bonds between protein and ligand in time scale

**Figure 11:**
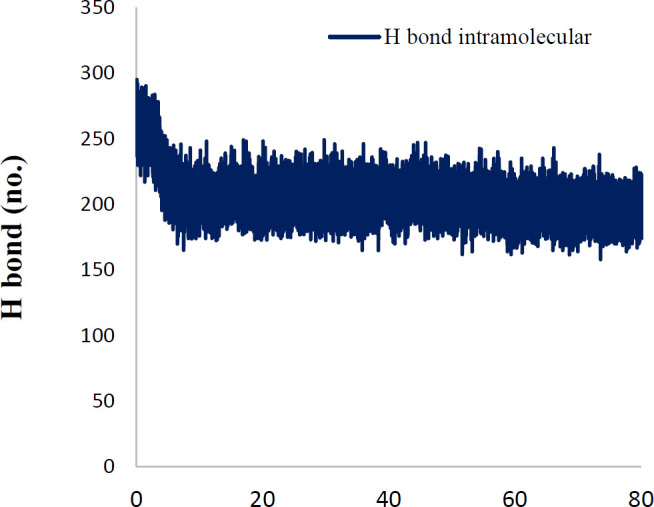
Number of intramolecular hydrogen bonds of protein in the time scale

**Figure 12:**
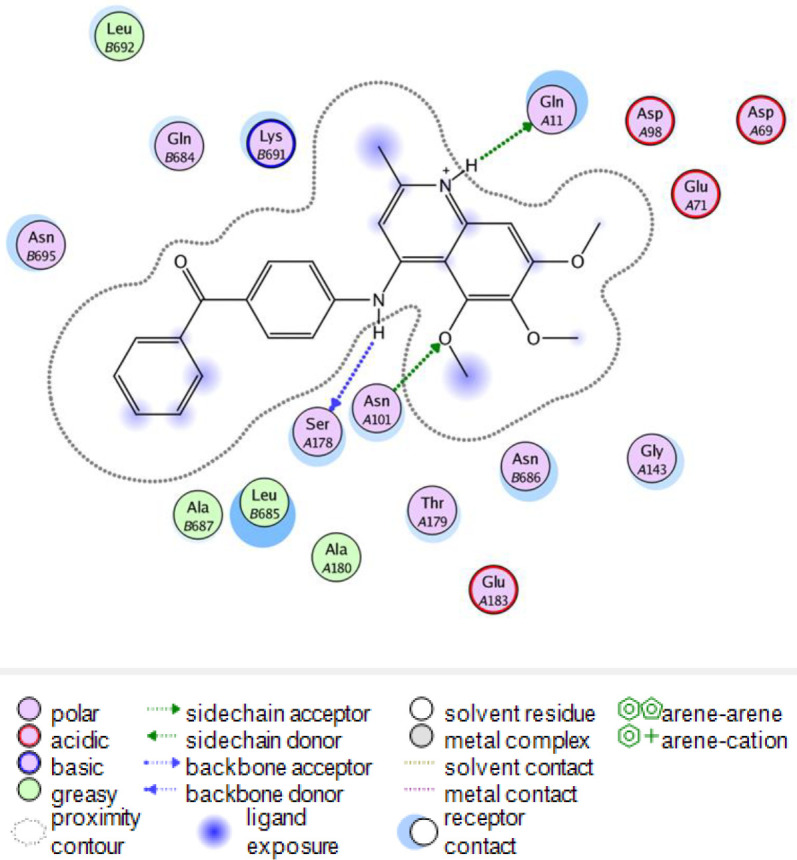
The 2D representation of the interaction between compound 7e in the crystal structure of tubulin (PDB ID: 4O2B) after 80 nsec molecular dynamic simulation using LigX in MOE

## Conclusion

Among the tested quinolines, **7e** and **7f,** possessing N-(4-benzoyl phenyl) and N-(4-phenoxyphenyl), respectively, exhibited strong cytotoxic activity against all four human cancer cells and also arrested cancer cells in the G2/M phase of cell cycle and proved to be tubulin inhibitor. In Annexin V binding staining studies compound **7e **induced cell apoptosis in a dose-dependent manner. Compound **7e** showed strong and selective antiproliferative activity in cancer cells and exhibited the lowest binding energy in molecular modeling study among all compounds. These results recommended that compound **7e **may be a promising lead compound for further development of anticancer agents.
